# Multiscale Characterization at Early Ages of Ultra-High Performance Geopolymer Concrete

**DOI:** 10.3390/polym14245504

**Published:** 2022-12-15

**Authors:** Mohamed Abdellatief, Hani Alanazi, Mohammed K. H. Radwan, Ahmed M. Tahwia

**Affiliations:** 1 Department of Structural Engineering, Faculty of Engineering, Mansoura University, Mansoura 35516, Egypt; 2 Department of Civil and Environmental Engineering, College of Engineering, Majmaah University, Al-Majmaah 11952, Saudi Arabia; 3 Department of Civil, Environmental and Mining Engineering, Faculty of Engineering, Computing and Mathematical Sciences, University of Western Australia, Crawley, WA 6009, Australia

**Keywords:** geopolymer concrete, gels, early compressive strength, durability, potassium hydroxide activator, freeze–thaw testing

## Abstract

The main obstacle of using geopolymer as a construction repair material is its slow strength development rate, which is the most significant attribute of an early-age opening for traffic and striking-off formwork. Geopolymer technology has recently attracted huge interest as an alternative to traditional cementitious materials with low environmental impact. Thus, this study investigates the feasibility of developing an ultra-high performance geopolymer concrete (UHPGC) with the aim of achieving high early-age strength. For this purpose, UHPGC mixtures activated with different potassium hydroxide molarities and aluminosilicate material types were developed and examined being cured with different curing temperatures. The early strength and durability of the UHPGC after 8 and 24 h were investigated. Experimental results revealed that the optimal mix design of UHPGC corresponds to a KOH molarity of 16 M and a 30% silica fume content. Furthermore, former mixture cured at 100 °C gave superior 8 and 24 h early strength values of 79 and 134 MPa, respectively. Moreover, a superior interaction of slag, silica fume, and activator solution at early age for UHPGC is revealed by the microstructural characteristics examined by a field emission scanning electron microscope (FESEM) with energy dispersive X-ray spectroscopy (EDS), Fourier transform infrared spectroscopy analysis, and thermogravimetric (TGA) techniques. It was also found that the compressive strength results and the results of the microstructure analysis are well coincided. The experimental results obtained in this study emphasize the feasibility of using developed UHPGC as an eco-friendly quick repair materials The development of one-part UHPGC as a quick, cost-effective, and high-strength product for all construction repair maintenance will lead to huge improvements in the structural capacity and durability of structural components.

## 1. Introduction

Many prominent characteristics of concrete made it the most utilized building mate-rial in the construction industry. With the vast development and inflated demand on concrete, several shortages along with severe environmental awareness have been raised. In addition, the volume of traffic has increased in urban areas all over the world, which has led to a significant increase in the heavy loads on roads and bridges. As a result, the bridge components, including its connections and concrete pavement, have presented different types of deterioration, and therefore, it needs to be repaired. Some requirements must be met in the process of maintenance and repair, including quick accomplishment, and gaining early strength, and it must also be environmentally friendly [1,2,3,4]. Due to the drawbacks of traditional cement-based concrete as a repair material, geopolymer technology has emerged as a good alternative to replace traditional concrete. Additionally, Portland cement concrete (PCC) has been regarded as the most widely utilized construction material worldwide [5,6,7]. However, PCC production process is well known as one of the processes that involve significant environmental impact. In this context, it is reported that cement production accounts for 5–8% of anthropogenic worldwide CO_2_ emissions [8,9]. In addition, it is well known that Portland cement (PC) is one of the most energy-intensive materials during the manufacturing process [10,11]. In an attempt to overcome these challenges, several studies have been conducted to develop less harmful and more ecologically friendly substitute binders [12,13,14]. As a result, the development of geopolymer as a potential substitute for PCC has received a great attention in recent decades [15,16].

Geopolymer system consist of aluminosilicate material (ASM) with fine particle sizes close to PC and act as a source of silicate (Si) and aluminium (Al), which in turn react in the presence of an alkaline solution. Slag (GGBS), fly ash (FA) [13,17], and metakaolin (MK) are common ASMs for geopolymerization. For activation, high alkali-materials such as sodium hydroxide and sodium silicate [18,19] are needed to complete the geopolymerization process. In the presence of the alkaline solution, ASMs are dissolved, resulting in repeating geopolymeric Si-O-Al-O interactions in amorphous form, which is the formation process in geopolymer technology. These reaction products are composed of a three-dimensional amorphous aluminosilicate network, with C(N)-A-S-H gels as the main products of this reaction in geopolymer concrete (GPC). This product has different characteristics compared to C-S-H gel, which is the main product of PCC [20,21,22]. Moreover, geopolymer presented comparable/improved mechanical performances with superior durability properties compared to PCC.

GPC could be developed to have excellent characteristics compared to PCC, namely superior acid, and sulfate resistance [22], elevated temperature resistance [23], abrasion resistance, and higher strength. In this regard, Rovnank [24] studied the influence of curing temperature and duration on dense GPC. It was observed that increasing the curing temperature accelerates the geopolymerization process, resulting in enhanced early strength. Contrarily, Hassan et al. [25] tested GPC curing at ambient temperatures, though, room-temperature curing was impractical. Hence, heat curing is necessary for GPC production. It is concluded that low curing temperatures slowed the geopolymerization process, which decreased the mechanical properties of GPC.

There has also been a lot of progress in GPC, where some recent studies have produced ultra-high-performance geopolymer concrete (UHPGC) that has the same strength and high packing density as cement-based ultra-high-performance concrete (UHPC) [26,27,28]. Aydin and Paradan [29] developed a UHPGC with a compressive strength of 200 MPa at 28 days of curing. Sodium hydroxide and sodium silicate were employed to activate ASMs under steam treatment, improving the mechanical properties of the geopolymer samples. Ambily et al. [19] developed UHPGC under ambient conditions and attained an improved mechanical performance, particularly its compressive strength. Even though both studies used GGBS and silica fume (SF) as ASMs, the impact of SF on early-age strength was not investigated. Wetzel and Middendorf [30] synthesized a UHPGC using SF and MK as a partial substitute of GGBS to enhance the workability of the UHPGC with low water-to-binder ratios. Their results indicated the possibility of a balance between hardened behavior and corresponding fresh characteristics. It was also reported that UHPGC achieved an ultimate compressive strength of 178.6 MPa when 12.5% SF was adopted, though compressive strength reduced with the increase of SF content up to 15%. J.C. Lao et al. [31] also produced a UHPGC with a compressive strength of up to 222 MPa using GGBS, SF, and FA with the incorporation of 4% steel fiber.

On the other hand, Kathirve and Sreekumaran [32] introduced a reactive powder concrete approach into the development of UHPGC. The results revealed that the incorporation of 30% of SF has a significant impact on mechanical property development. Unfortunately, to date, UHPGC microstructural investigations are scarce. Wan et al. [33] investigated the effects of GGBS, FA, sodium silicate modulus, Ca/(Al + Si), Si/Al, and SF on UHPGC strength development using scanning electron microscopy (SEM) and X-ray diffraction (XRD) based on micromorphology and reaction products. Dolomite, zeolite, and C-(N)-A-S-H gel are important reaction products, according to SEM and XRD investigations. Tahwia et al. [34] and Cai et al. [16] investigated the microstructure of the UHPGC matrix. It was concluded that the microstructure of the UHPGC matrix is compact, and there is strong bonding between the geopolymer gel and fine aggregate. It is well acknowledged that increasing the dissolution of source materials leads to a higher amount of C (K)-A-S-H gel, which improves the mechanical characteristics of the concrete.

Meanwhile, UHPGC is a modern form of concrete with superior strength and durability. To date, there is a considerable demand for effective and long-lasting building materials that exhibit adequate resistance to deterioration due to freeze–thaw cycles, delamination of concrete cover, deck cracking, and reinforcing steel corrosion. Therefore, considering the limited studies done thus far, UHPGC’s practical uses are still restricted, despite its promising mechanical characteristics at room temperature. Only a few studies examined the early-age properties of UHPGC, despite the fact that early-age properties must be known before UHPGC is utilized as a rapid repair material for concrete constructions and pavements, allowing a quick re-opening to traffic.

Therefore, this study aims to evaluate the effect of different potassium hydroxide molarity, binder materials, and curing temperature on the early compressive strength, durability, and microstructure of UHPGC system. In this experimental study, first, the optimum potassium hydroxide molarity and SF content were determined. Then, the effect of the curing condition on the early compressive strength of geopolymer mixtures with optimum parameters was investigated. Finally, the microstructural characteristics and chemical properties of the prepared UHPGC mixtures were examined.

## 2. Materials and Experimental Methods

### 2.1. Materials

**Figure 1:**
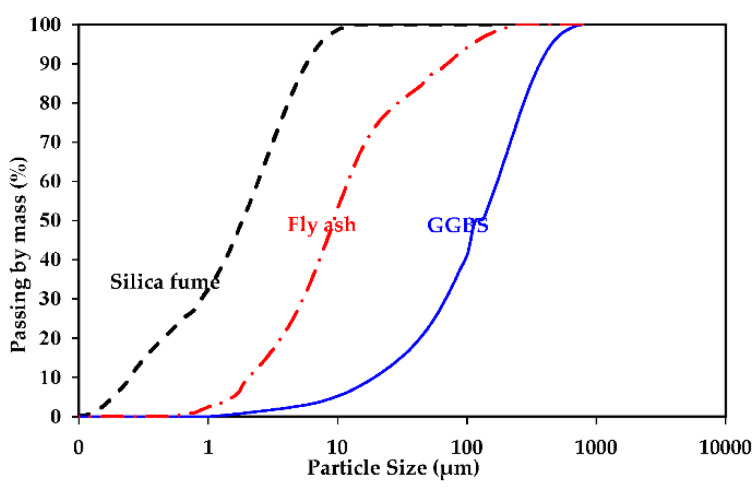
Particle size distributions of ASMs.

**Figure 2:**
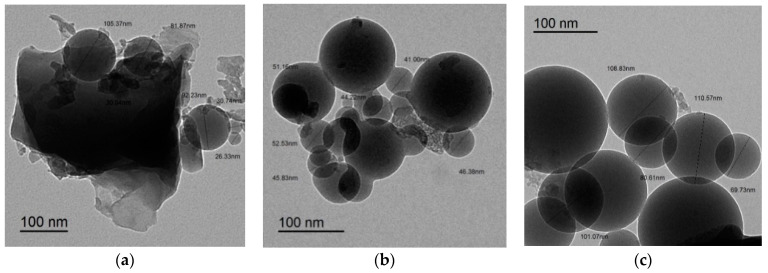
TEM of ASM, (**a**)GGBS; (**b**) SF; (**c**) FA.

The primary binder materials that were used in the current study were slag, FA, and SF. To increase early compressive strength and reduce capillary porosity, the grain size distributions of all binders were evaluated using laser granulometry. Based on these findings, an optimum packing density was estimated using a method reported by Wong et al. [35]. Therefore, SF is a particularly effective way to increase packing density. SF has a maximum silica concentration of 98.86%, a specific gravity of 2.29 g/cm^3^, and a specific surface area of 17,500 m^2^/kg. Meanwhile, the GGBS has a specific gravity of 2.90 g/cm^3^ and a specific surface area of 396 m^2^/kg. The FA has a specific gravity of 2.2 g/cm^3^ and 90% of FA was passed through 67.5 µm. The particle size distribution in terms of intensity for GGBS, SF, and F was performed, and their distribution curves are shown in Figure 1. The main chemical compositions of GGBS, FA, and SF were listed in Table 1. For more characterization of the particles’ shape of ASMs, the transmission electron microscope (TEM) has been implemented, as shown in Figure 2. It was observed that SF is an ultra-fine spherical particle; however, FA has a coarser spherical particle. It can also be observed in Figure 2 that GGBS has an irregular shape with a larger diameter compared to both SF and FA.

Natural sand with two particle size ranges, including grade I (0.125/0.6) mm and grade II (0.80/2.36) mm, was used as fine aggregate. The specific gravity and water absorption of the used sand were 2.65 g/cm^3^ and 0.50%, respectively. The fineness modulus of combined sand (grade I and II) was 2.71. These used sands are following BS EN 12620. The alkali activator solution consisted of waterglass (Na_2_SiO_3_) solution, potassium hydroxide flakes (KOH), and tap water. The mass ratio of SiO_2_ to Na_2_O of the Na_2_SiO_3_ solution was 2.61, with chemical compositions of 30.3% SiO_2_, 11.6% Na_2_O, and 58.1% water. The specific gravity of the combined alkali activator was about 1.6 g/cm^3^, while that of Na_2_SiO_3_ and KOH was 1.65 g/cm^3^ and 1.38 g/cm^3^, respectively.

**Figure 3:**
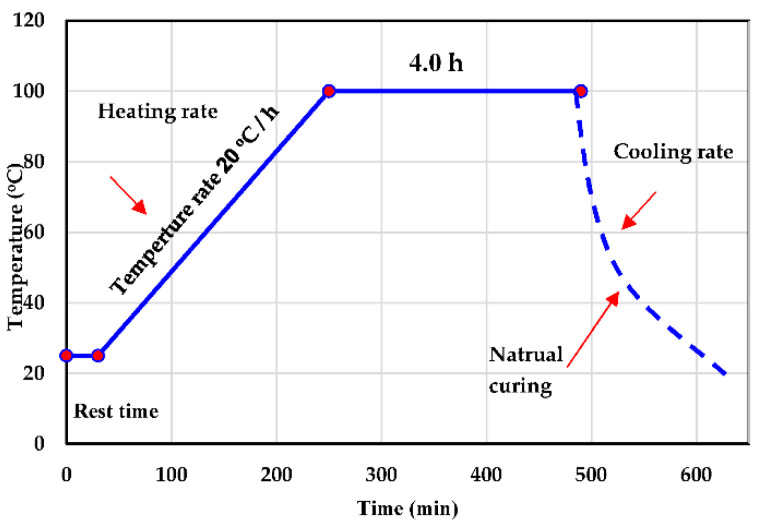
Schematic temperature exposure regimen.

The main criteria for this work are to control the early strength and durability of geopolymer concrete in terms of the ASM types, potassium hydroxide molarity, and curing temperatures. Table 2 summarizes the parameters used for early-age compressive strength development. Two series of UHPGC mixtures were generated based on GGBS. The first series, SF, was introduced as a partial part of ASMs in 10% steps (by vol.), while the second 10% FA was added to ASM (by vol.) in the presence of SF. In addition, three molarities of KOH were taken into consideration: 12 M, 14 M, and 16 M. According to previous studies, frequent molarities that generate high strengths vary from 12 M to 16 M; hence, these concentrations were chosen [19,34]. Furthermore, three curing temperature degrees were considered in this study, including 60 and 100 °C, as well as ambient curing (25 ± 2 °C), as shown in Figure 3 [36,37].

### 2.2. Samples Preparation

The UHPGC combinations were proportioned based on the previous work conducted on UHPGC mixtures [38]. Table 3 shows the mixed proportions. The alkali activator was formed by allowing it to cool to room temperature for one day before UHPGC production [27]. An alkali activator /ASM ratio of 0.36 was selected after conducting several trial tests to achieve the desired strength and to attain adequate workability. All mixtures were prepared with different ratios of ASMs. The control mixture was designed with GGBS solely as ASM and labeled as “G1”. Series 1 was designed by replacing GGBS with SF by 0%, 7.5%, 15%, 22.5%, and 30% of total ASM (by vol.). Additionally, Series 2 were created as that of Series 1; however, 10% FA of total ASM (by vol.) was adopted.

Dry components were firstly mixed in a laboratory Hobart mixer for 120 s at a low speed (90 rpm) until a homogeneous grey–light color combination was achieved. After that, the alkali activator was gradually added to the mixture and mixed for another 100 s. After the entire components were incorporated, the mixture was mixed at high speed (182 rpm) for 60 s. To complete mixing phase of each designed mixture, around 6–8 min was required. Finally, all samples were cast in the molds and covered to prevent any moisture loss, then stored at room temperature for 4 h [32,39]. Upon demolding, some specimens were kept at room temperature until testing day. The remain specimens were treated under heat curing (Figure 3). A set of three specimens was inspected at 8 and 24 h for each of the mixtures.

### 2.3. Experimental Methods

#### 2.3.1. Fresh, Mechanical, and Durability Tests

The flowability of fresh UHPGC was assessed in accordance with ASTM C1437. Following ASTM C191, the setting times of fresh mixtures were measured. The setting time test was performed at 25–27 °C. The dry density of UHPGC was measured in accordance with ASTM C138. The compressive strength test was conducted at 8 and 24 h. The compressive strength of UHPGC specimens was tested using standard testing apparatus at a maximum load of 2500 kN. The compressive strength loading rate was 1.0 MPa/s. 50 mm cubic samples were used to assess the compressive strength. Additionally, the nondestructive test was employed to calculate the ultrasonic pulse velocity in accordance with ASTM C597-16.

For the measurement of the rate of absorption of water, ASTM C1585-04 was followed. Additionally, prism samples were prepared to assess the resistance of the mixture to freezing and thawing where the specimens were frozen and thawed. The tray was then filled with distilled water and coated with the plastic film per ASTM C666. The mass loss of sample weight results for each group were taken as the average of three samples after 25 cycles to determine the freeze–thaw values. The porosities of hardened UHPGC were evaluated using ASTM C642 to assess the compactness of its microstructure, which influences mechanical and durability performance. The oven-dried mass (W_1_), saturated surface–dry mass (W_2_), and immersed apparent mass (W_3_) of each mixture were determined. The water-permeable porosity of UHPGC mixtures is given by Equation (1), and the mean value was calculated for each mixture.

Porosity = {W_2_ – W_1_} ∗ 100/{W_2_ – W_3_} (1)

#### 2.3.2. Microstructure Analysis

The morphological characteristics of UHPGC matrices were analyzed using SEM. At various magnifications, SEM was performed on 0.5 mm slices of the ambient-cured material. EDS analysis was utilized to determine the elemental compositions of hardened UHPGC mixtures. In addition, the Fourier transform infrared (FTIR) spectrum was obtained using a Spectrum 400 FT-IR to characterize the presence of a functional group of hardened geopolymers. A differential scanning calorimeter was used to define the thermogravimetric (TG/DTG) curves of hardened powdered concrete. Analyses are conducted at a heating rate of 10 °C/min from 25 °C to 800 °C in a flowing nitrogen atmosphere. The mass loss was calculated while the sample was gradually exposed to temperatures up to 800 °C.

## 3. Results and Discussion

### 3.1. Flowability

**Figure 4:**
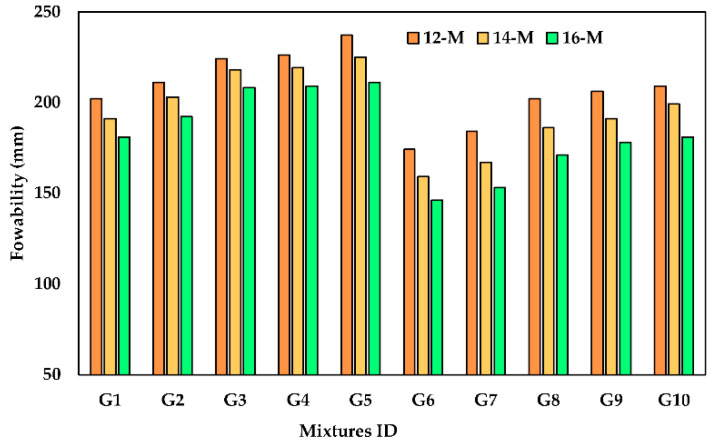
Effect of KOH molarity and ASM type on the slump flow diameter.

The influence of KOH molarity on the flowability of UHPGC mixtures with different kinds of ASM was carried out and presented in Figure 4. Generally, it was observed that UHPGC flowability increased when KOH molarity was reduced. This is because more water was being added to the mix, which acted as a lubricant and enhanced the spreadability of the mixtures. On the other hand, increasing KOH concentration raised pH and dissolution rate, which improved the geopolymerization process. So, higher KOH molarity increases viscosity and reduces combination flowability. This can be inferred from the finding that the H_2_O/SiO_2_ ratio decreases with increasing molarity solutions [40].

Moreover, GGBS exhibited an adverse effect on the flowability of UHPGC mixtures. For instance, while the GGBS amount was reduced from 100% to 70% at 12 molarity (G5), the flow diameter increased from 202 mm to 237 mm (17.32% improvement) was observed. While at 16 KOH molarity (G5), the flowability increased from 181 mm to 211 mm (16.57% improvement). On the other hand, the flowability of the UHPGC mixtures was reduced with the addition of 10% FA (Series 2), as shown in Figure 4. Compared to control mixture, the flowability of UHPGC containing 10% FA dropped by 13.86%, 16.75, and 19.33% at 12, 14, and 16 M, respectively. The reason for this reduction in the flow rate was that the FA particles made the mixture stickier, resulting in increased friction between gel particles. These results are consistent with the previous studies [41,42]. It is worth noting that previous investigations have demonstrated that geopolymer concretes with slump values above 200 mm have reasonable workability for casting structural elements such as beams and columns [43]. For workable GGBS-based GPC, Yusuf et al. reported H_2_O/Na_2_O molar ratios in the range of 18.9 to 23.1 [44]. The H_2_O/Na_2_O molar ratios reported by Juengsuwattananon et al. [40,44,45] were in the range of 7.5 to 27.5. The H_2_O/Na_2_O ratio for all the mixtures in this investigation presented within this range (Table 4). In these literature works, it was reported that a higher H_2_O/Na_2_O molar ratio influenced compressive strength adversely. On the contrary, enhanced workability can be achieved with a higher H_2_O/Na_2_O molar ratio. These conclusions agree well with results obtained in this study.

### 3.2. Setting Time

**Figure 5:**
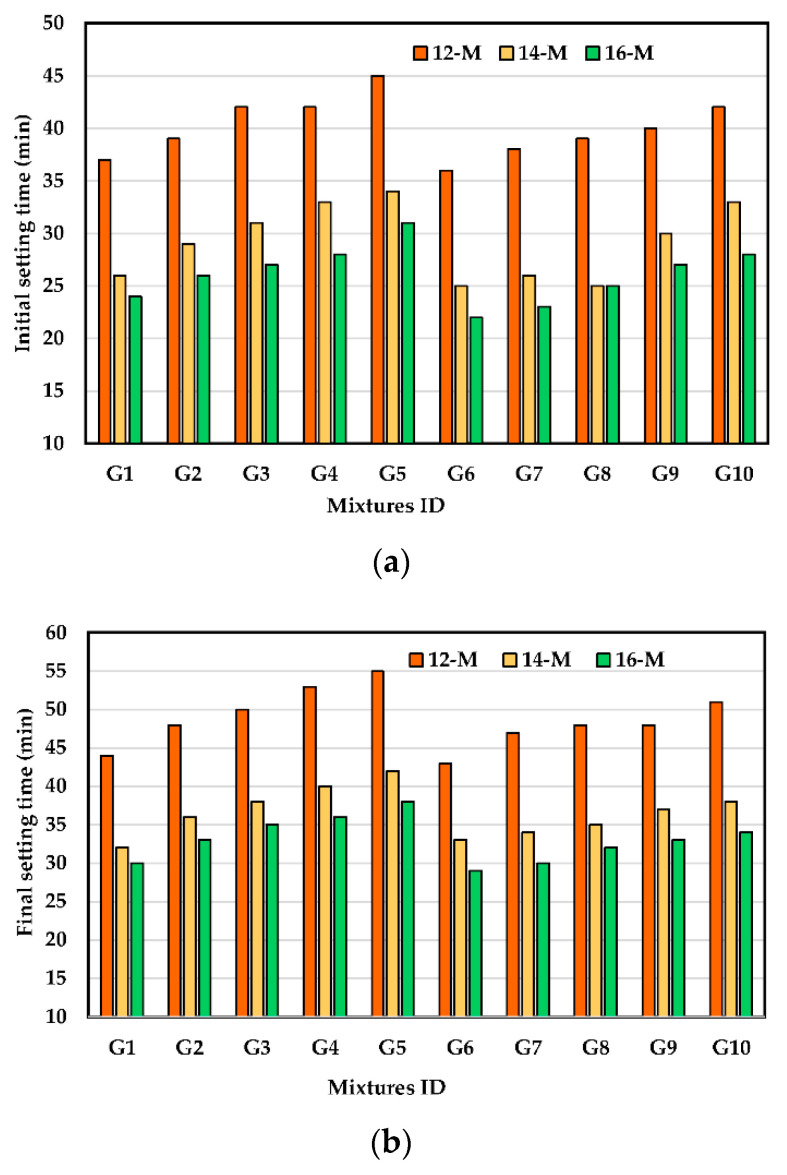
Effect of KOH molarity and ASM type at room temperature on ST of the UHPGC. (**a**) Initial setting time. (**b**) Final setting time.

In this study, the initial and final setting times (ST) of UHPGC changed in the range of 19 to 45 and 26 to 56 min, respectively. UHPGC typically sets in this condition quickly due to the rapid rate of chemical reaction at ambient temperature. The influence of KOH molarity on the initial and final ST of UHPGC with different combinations of ASM is shown in Figure 5. Increasing the molarity of the KOH solution shortened the initial and final ST for the UHPGC mixtures. This is consistent with the findings of prior studies [30,31] and could be attributed to the reduction in water content. For example, increasing the molarity from 12 M to 16 M causes a reduction in the initial ST from 37 min to 24 min and the final ST from 44 to 30 min. The difference between the initial and final ST also decreased with the increase in KOH molarity in the UHPGC. This could be because increasing the molarity of KOH accelerated the rate of geopolymerization and made the precursors of aluminosilicate dissolve faster [30,46,47]. This behavior could be explained by the fact that at lower KOH solution molarities, more calcium ions are available to react with water to form the C-S-H and C-A-H gels, whereas at higher molarities, more K+ and OH-ions are present, which enhances the dissolution rate of the alumino-silicate precursors and reduces the leaching of the calcium ions [40,41]. Conversely, increasing the SF level in UHPGC mixtures causes both the initial and final ST to increase. As an example, for UHPGC mixtures, increasing SF content as ASM replacement from 7.5% to 30% increases the initial ST from 37 min to 45 min (Figure 5a) and increases the final ST from 44 min to 53 min at 12 KOH molarity (Figure 5b). This is because the alkali activator solution likely accelerates the geopolymer reaction by increasing the speed of dissolution of ASM as reported [19,34]. It is worth observing that the ST had the same trend of flowability [32]. The effect of SF on the setting time of geopolymer mixture can be attributed to a partial dissolution of silica fume increasing the Si/Al ratio.

It is also noticed that, compared to the control mixture, UHPGC containing 10% FA exhibited slightly lower initial and final ST. The initial ST reduced from 24 min to 22 min (Figure 5a), and the final ST reduced from 30 min to 29 min at a high KOH molarity (Figure 5b). It is evident that the initial and final STs of UHPGC have different parameters depending on the type of ASM used. It is also proven that at the same KOH molarity, SF mixtures have the longest ST while GGBS mixtures have the shortest initial and final ST. As a result of the findings, it can also be said that UHPGC-containing SF mixtures are better suited for site applications compared to solely control UHPGC (GGBS) mixture since they are set up more quickly at room temperature. The earlier findings concurred with several previous investigations [9].

### 3.3. Early Compressive Strength

**Figure 6:**
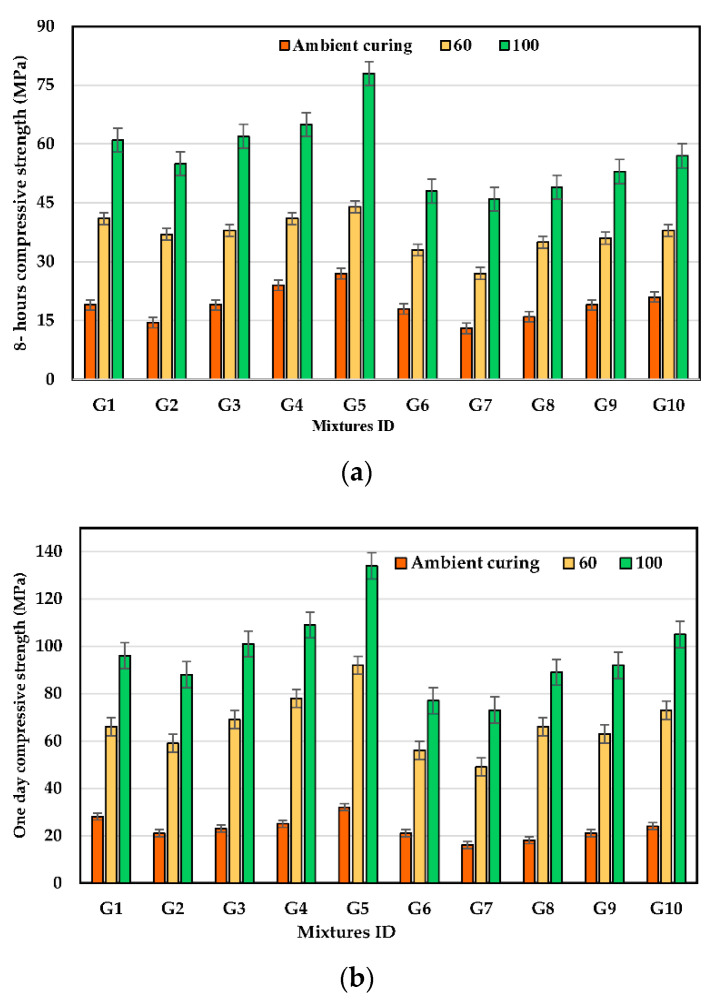
Effect of curing temperature and ASM type on early CS at KOH molarity of 16. (**a**) CS after 8 h; (**b**) CS after 24 h.

Figure 6 illustrates the influence of the curing temperature and the ASM on the early compressive strength (CS) of the UHPGC at 8 and 24 h at KOH molarity of 16. The curing temperature significantly affects the CS of UHPGC; the CS of all UHPGC mixtures, notably those containing SF, is improved when the curing temperature is raised from room temperature to 100 °C. According to Figure 6a, CS at 8 h was increased for the control mixture (G1) by 115.8% and 221.3%, while 62.9% and 188.8% for mixtures containing 30% SF (G5) at 16 M, when cured at 60 °C and 100 °C, respectively, as compared to ambient curing. Similarly, CS at 24 h increases for the control mixture by 135.7% and 242.85%, while it increases by 187.5% and 318.75% for G5 when cured at 60 °C and 100 °C, respectively. This pattern can be attributed to the improvement in the geopolymerization process as polymer chains lengthen, which enhances the rate at which alumino-silicate minerals dissolve because of rising curing temperatures. This result is consistent with earlier studies [19,34].

Figure 6 also shows that by increasing curing temperatures, the early CS of concrete increases. Therefore, high early compressive strengths at 100 °C were 79 MPa after 8 h of casting, while high compressive values after 24 h of casting were 134 Mpa at 16 KOH molarity. The reason for this high early strength is the proper choice of raw material and graduation of particles to achieve a high packing density of materials and enhance the geopolymerization process. It is likely that the polymerization reaction process and the hydration process were enhanced due to the inclusion of SF, which increased the Si/Al ratio in the mixtures, leading to an increase in the early compressive strength. In addition, the results of the early strength treated under ambient conditions were very low, as the rate of maturation was very weak (slowing of the polymerization process), and the maximum compressive strength was reached at 8 h and 24 h by heat curing. According to Alanazi et al., the molar ratio of silicon dioxide/sodium oxide plays a crucial role in the creation of a geopolymer. A silicon dioxide/sodium oxide molar ratio of 1.0 resulted in the shortest curing time and the maximum early strength after 24 h [37]. The molar ratio of this study is presented in Table 4.

**Figure 7:**
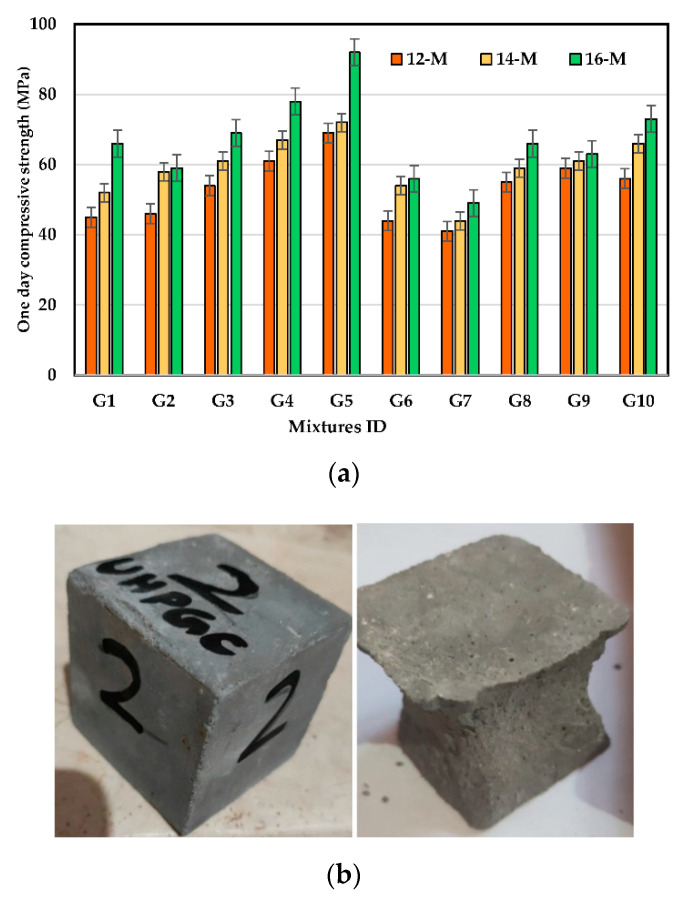
The performance of UHPGC at early ages. (**a**) Effect of KOH molarity and ASM type on 24 h CS at curing heat 60 °C; (**b**) UHPGC before and after testing at 24 h.

Figure 7a illustrates the impact of KOH solution molarity on the early CS of UHPGC with different kinds of ASMs at curing temperatures of 60 °C. According to the test results, the KOH molarity has a significant impact on the CS of all UHPGC combinations. By comparing the mixtures of 14 M and 16 M to 12 M, there was an increase in CS for the control mixture by 15.5% and 46.5%, respectively. The increase in KOH molarity may expedite geopolymerization by accelerating aluminosilicate dissolution. Contrarily, the addition of FA reduces the early compressive strength at all alkali concentrations compared to when solely SF was added.

It is worth noting that Karimipour et al. [47] found that the mechanical and fracture properties of UHPGC decreased significantly with the use of 10% SF and then improved after that by adding more than 10% SF. Therefore, the present work is aligned with these results. The explanation for this phenomenon is that SF has a high percentage of silica in its composition (98.8%), which led to an improvement in the polymerization process and the filling of the micro-pores. Contrastingly, when 10% of FA was incorporated as a percentage of slag in all mixtures, the early compressive strength declined. Figure 7b shows the failure shape of the UHPGC samples at 24 h. Based on visual observation, a true failure occurred, and the core of specimen was found to be well-filled. It worth mentioning that no obvious cracks were observed prior or after UHPGC was heat cured at high temperatures.

In recent studies, the production of early compressive geopolymer matrices has mostly focused on optimizing the parameters and the components of the matrices to achieve the desired strength. In general, the early compressive strength of geopolymer composites is affected by the temperature of curing, alkali activator solution type and ratios, and binder type and content. Therefore, the high early compressive strength of geopolymer matrices of the control mixture, the highest compressive strength in this study, and the highest early strength published in earlier references are shown in Table 5. The main reason for the increase in early compressive strength is the heat treatment process, which works to improve the geopolymerization process and the formation of additional phases of the C-(N)-A-S-H. This leads to better porosity, which works to strengthen the microstructure of the mixture, which will be explained in Section 3.8.1. It is worth noting that the use of UHPGC as a repair material will lead to huge improvements in the structural capacity and durability of structural components. In addition, this study investigated both ambient curing and heat curing for early compressive strength. The ambient curing for UHPGC can be used for pavement repair. On the other hand, the high early strengths of UHPGC at heat curing can be used for special cases and other future applications.

### 3.4. Ultrasonic Pulse Velocity Test

**Figure 8:**
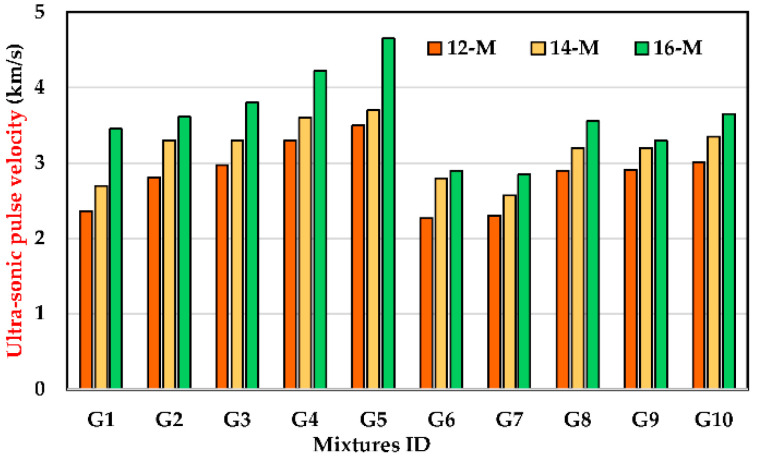
Effect of KOH molarity and ASM type on ultrasonic pulse velocity of the UHPGC at curing heat 60 °C.

**Figure 9:**
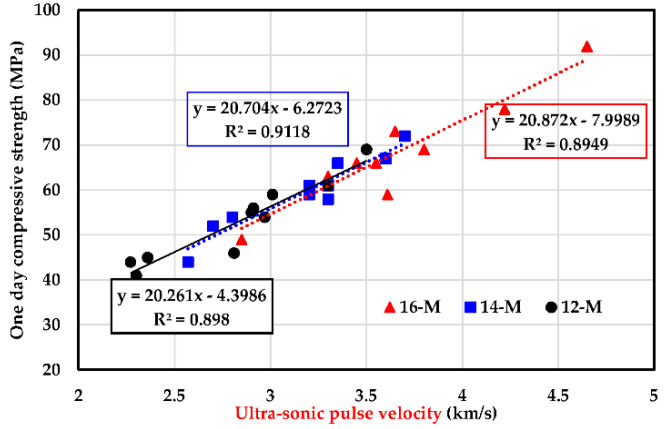
Comparison of 24 h CS with ultrasonic pulse velocity at 60 °C of curing temperature.

The variation in ultrasonic pulse velocity of UHPGC mixtures at 24 h for different KOH molarity and ASM types at curing temperatures of 60 °C is shown in Figure 8. The ultrasonic pulse velocity increased as the alkali molarity and SF increased in all the UHPGC mixtures. For instance, at a KOH molarity of 16, the inclusion of 0% to 30% of SF enhanced the UPV values by 4.63% to 34.7% compared to G1. On the other hand, the inclusion of 10% FA reduced the ultrasonic pulse velocity value by 9.6%, 12.5%, and 15.96% at 12, 14, and 16 M, respectively, compared to G1. This demonstrates the dual effect of SF which contribute to filling the micro pores in the mixture and developing of additional silicate gels due to the pozzolanic reaction. Altogether, the addition of SF tends to densify the UHPGC mixture, which in turn facilitates the travel of waves in less time, especially at higher temperatures. The results are consistent with compressive strength and show that UHPGC with SF performs better than GGBS alone. From another perspective, the increase in the ultrasonic pulse velocity value in the case of a high concentration of alkali activator might be related to the enhancement of the geopolymerization process, especially in thermal environments [42]. On the other hand, these parameters make the structure nonporous and compacted, which accelerates the polycondensation reaction as part of geopolymerization [37]. All ultrasonic pulse velocity results were shown in Figure 9 to establish a correlation with the early compressive strength of UHPGC.

### 3.5. Relation between the Compressive Strength and Dry Unit Weight at Early Age

**Figure 10:**
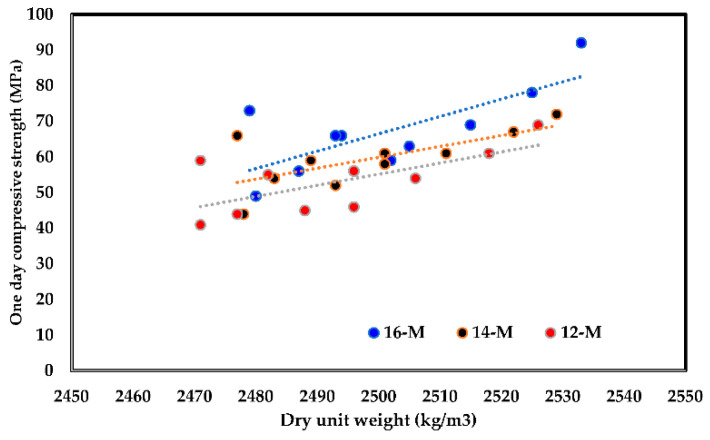
Relation between the CS and unit weight of at different KOH molarity under curing heat 60 °C.

The relationship of compressive strength and dry unit weight of the UHPGC mixtures with different types of KOH molarity at 24 h are shown in Figure 10. The total dry unit weight of UHPG varied between 2471 and 2533 kg/m^3^. An increasing trend can be observed for both of compressive strength as well as the unit weight, which also magnified when a higher amount of SF was incorporated. The higher compressive strength of UHPGC mixtures cured by the addition of 30% SF indicates that the important role of SF properties such as particles fineness and high surface area is beneficial to the strength development of geopolymer. This observation is also in good agreement with previous studies [41,48]. Additionally, the unit weight of UHPGC mixtures containing 10% FA had a slight decline, which demonstrated that the trend of the early compressive strength correlates well with that of dry unit weight.

### 3.6. Water Sorptivity

**Figure 11:**
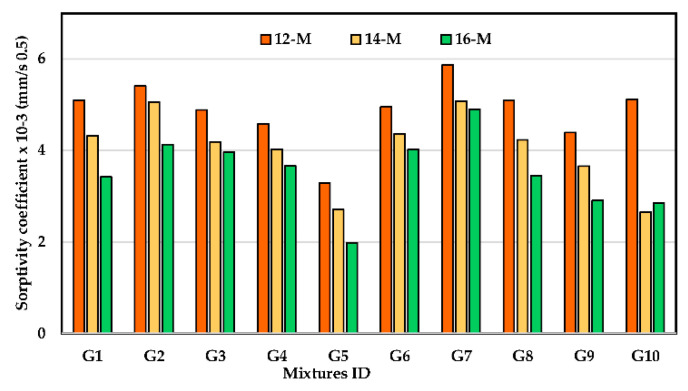
Effect of KOH molarity and binder type on sorptivity coefficient of the UHPGC at curing heat 60 °C.

Sorptivity is one of the most widely used permeability tests. This test mostly relies on how water enters the concrete through continuous pores. Figure 11 illustrates the water sorptivity coefficient of studied UHPGC mixtures. As anticipated, the combined effects of SF incorporation and increased KOH molarity resulted in a decrease in the sorptivity coefficients of the UHPGC. The maximum sorptivity was measured in the case of G7 with 12 M, whereas the minimum sorptivity was noted for G5 at a KOH molarity of 16 as 2.10 × 10^−3^ mm/s^0.5^. It was observed that the comparable percentages of decrease brought on by SF incorporation (G5) were 32.2%, 35.6%, and 50.2% for UHPGC activated with KOH molarities of 12, 14, and 16, respectively, compared to G1. The comparable amounts of decrease owing to SF incorporation and 10% fly ash were 12.9% (G7), 13.78% (G8), and 15.65% (G9), respectively. This reveals that UHPGC capillary water absorption relies on matrix quality and binder type. The finer pores and lower porosity reduce the capillarity [36,38]. This prevents water from entering and causing concrete deterioration and reinforcement corrosion. In addition, minimizing sorptivity tends to reduce the entry of chloride- or sulfate-containing water into concrete, which can be destructive.

In Table 6, the porosity revealed a similar trend in the compressive strength with the increase in SF dosage. For instance, the highest porosity is recorded for UHPGC mixture containing 10% FA (G7). It was also observed that by adopting 30% of SF as a binder replacement (GGBS) along with a high KOH molarity, higher early strength and lower porosity could be achieved.

### 3.7. Relation between the Freeze–Thaw Cycles and Compressive Strength

**Figure 12:**
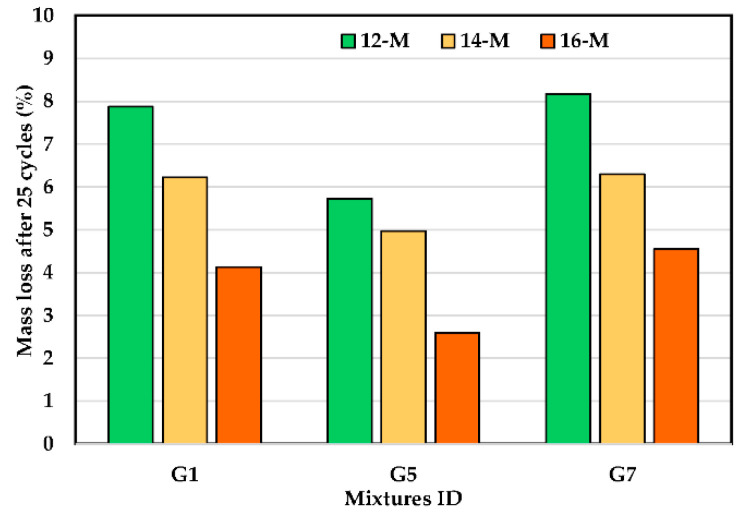
Effect of KOH molarity on freeze–thaw cyclic of the UHPGC at curing heat 60 °C.

Figure 12 presents the weight loss results for three UHPGC mixtures following 25 freeze–thaw cycles in accordance with ASTM C666. To avoid repetition in this study, three series of UHPGC mixtures were chosen: G1 (control mix), G5 (the best mix had 134 MPa), and G7 (high porosity, 3.63%). It was found that the change in alkali activator concentration affected the durability of the samples. For instance, the control mixture experienced mass loss at 12 KOH molarity (7.88% decline), whereas the mass loss was reduced to 6.22% and 4.13% at 14 M and 16 M. In addition, the proper percentage of SF also reduced the mass loss of the geopolymer concrete samples. In other words, the G5 specimens achieved the lowest mass loss at 12, 14, and 16 M compared to the control mix, which was 27.4%, 20.2%, and 37.1%, respectively. In contrast, the G7 mixture experienced the highest mass loss compared to the other mixtures at similar KOH molarity. The results suggest that the UHPGC with fly ash is less frost resistant than one with SF or GGBS. These results are consistent with previous studies [37,53]. Substantially, under ASTM C666, all mixtures with 16 M offer the best durability when compared to the other combinations because they lose less than 5% of their weight. The concrete surface pitting caused by freeze–thaw activity was the main cause of the mass loss that was seen. However, the findings show that after just 25 cycles, there has been a considerable loss of bulk, especially at 12 KOH molarity. The control mix and G7 in its current mix appear to be less durable than cement concrete. However, a G5 mix with the right amount of SF is more resistant to freezing and thawing than cement concrete.

### 3.8. Microstructure of UHPGC

#### 3.8.1. Scanning Electron Microscopy (SEM) and EDX Analysis

**Figure 13:**
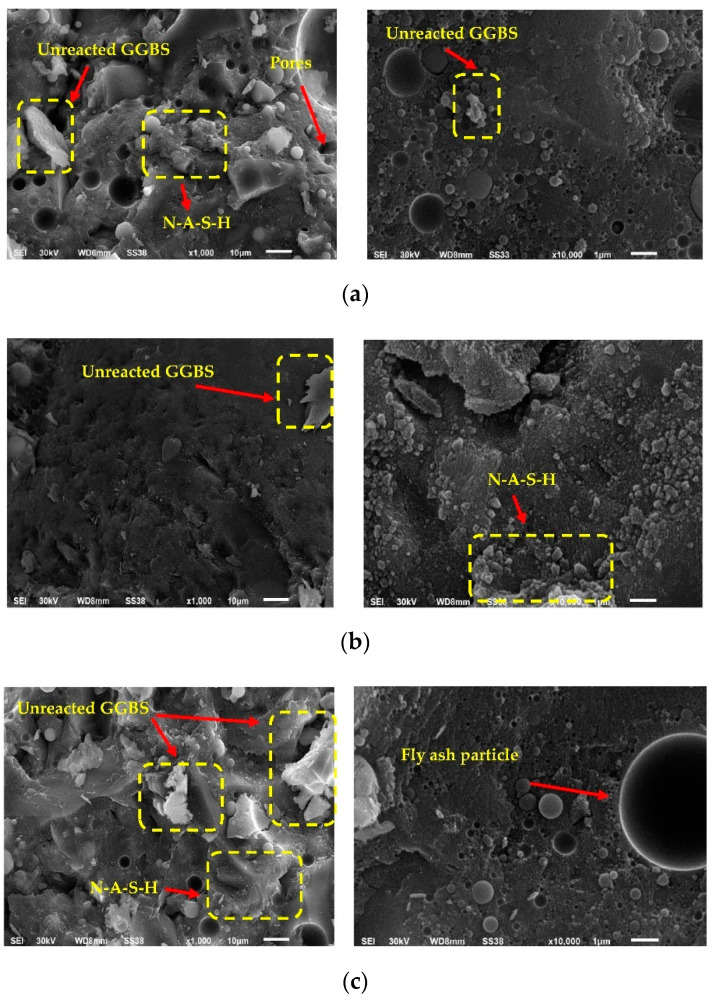
SEM images of UHPGC mixtures. (**a**) G1 (control mix) at 10 microns and 1-micron magnification; (**b**) G5 at 10 microns and 1-micron magnification; (**c**) G7 at 10 microns and 1-micron magnification.

SEM was used to examine the influence of ASM type on the microstructure of geopolymer concrete at early ages as presented in Figure 13. To discuss the microstructure of UHPGC, the control mixture, the optimum mixtures (G5), and G7 were observed. The curing regime (heating to 60 °C) and the KOH molarity (16 M) are the same in these mixtures. Overall, it was revealed that the mechanical behavior of geopolymer concrete was connected to its microstructure [46]. As shown in Figure 13, a significantly denser microstructure was observed with increasing the content of SF compared G1. Furthermore, the system with a high Si/Al ratio contained high C-A-S-H gel, resulting in the production of UHPGC mixes with low porosity [54]. The presence of SF in the UHPGC composites plays a key role in enhancing the microstructure of the UHPGC matrix, which is positively reflected on its mechanical characteristics as determined by SEM imaging (Figure 13b). The amorphous nature of SF, as well as the ability to enhance the packing density of the UHPGC composite, may have contributed to the improvement in the microstructure and other characteristics of UHPGC. On the other hand, using GGBS alone in the geopolymer system led to the generation of a reaction product (C-S-H gel) in addition to the geopolymer (N-A-S-H) gel (Figure 13a). It was likely the reason for the high calcium content in the GGBS composition, as mentioned in Table 4. The microstructure of G5 was found to be denser than that of the control mixture (G1) and G7 (Figure 13c). This is in line with the trend of compressive strength.

**Figure 14:**
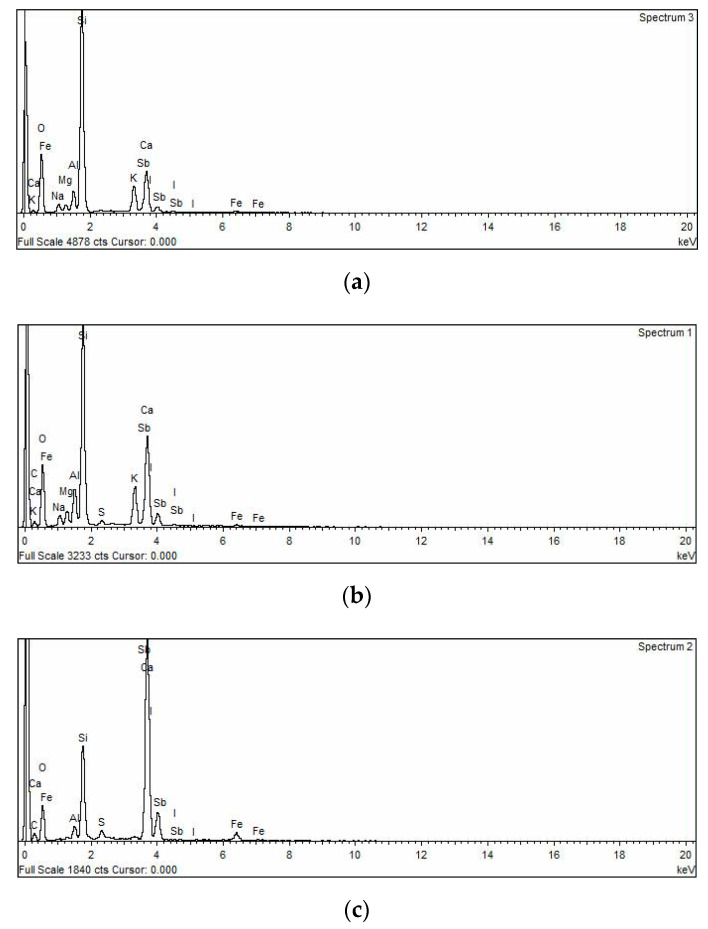
EDX analysis of UHPGC mixtures, (**a**) G1; (**b**) G5; (**c**) G7.

On the other hand, the results of EDX analysis for G1, G5, and G7 mixtures at 24 h are shown in Figure 14. As shown by the EDX analysis, the element content changes as a result of utilizing SF as a partial substitute for ASM. Sodium (Na), silicon (Si), magnesium (Mg), calcium (Ca), potassium (K), aluminium (Al), oxygen (O), and iron (Fe) were the principal fundamental constituents of the geopolymer matrix. The figure also demonstrates that, in comparison to UHPGCs containing 10% FA and the control mixture, UHPGCs including SF had higher combined concentrations of silicon, aluminium, and calcium (the primary components of C-A-S-H and C-S-H hydration products) [34]. In addition, the increased maximum intensity of Si and Mg with the introduction of 30% SF demonstrated the existence of akermanite (Ca_2_Mg[Si_2_O_7_]), explaining the enhancement of compressive strength. In contrast, the inclusion of 10% FA leads to the absence of some elements such as Mg, which is related to the low compressive strength.

#### 3.8.2. Thermal Analysis

**Figure 15:**
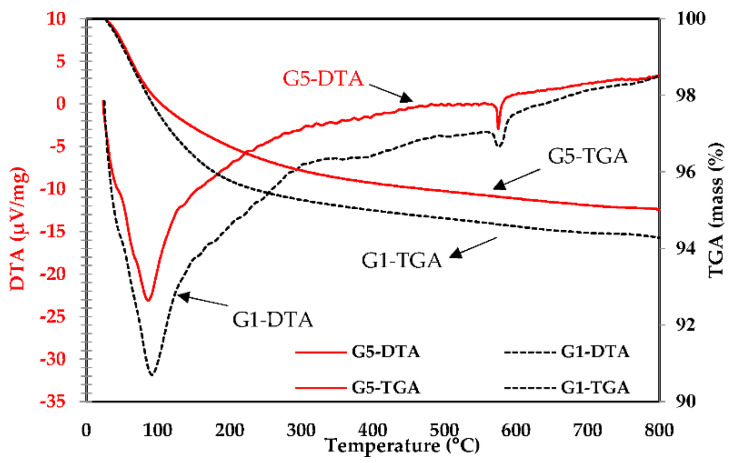
Weight loss and derivative thermogravimetric (DTA) curves of UHPGC mixtures.

Figure 15 presents the mass loss and derivative thermogravimetry (DTA) curves for the UHPGC mixtures. As can be seen from the curve, all mixtures have a similar trend of losing weight. However, they have varied mass loss ratios at each variable temperature range, indicating that the quantity of a material changes at each reaction stage. In general, the curve has three main temperature ranges. The first has a temperature range of 20 °C to 200 °C, indicating that the C-A-S-H phases and physically bonded water have dehydrated [55]. The evaporation of free and physically bound water, as well as the hydration of C-A-S-H, was related to the initial weight loss peak of the TGA/DTA curves at around 100 °C, as shown in the curve. The first weight loss peak resulted from the dehydration of the geopolymer gel up to approximately 350 °C. The second mass-loss peak occurred between 400 °C and 600 °C due to the dehydroxylation of calcium hydroxide, and the final peak occurred up to 800 °C due to the decarbonation of calcium carbonate [16,53]. The TGA/DTA results indicated that the increment of SF content could reduce the mass loss resulting from the decomposition of calcium carbonate at elevated temperatures. The control mix loses 6% from its weight; however, G5 maintains more than 95% of its weight. On the other hand, it is observed that mixtures with a high Si/Al ratio had the lowest weight loss, while mixtures with a low Si/Al ratio had the highest mass loss (Table 4). The reason for this phenomenon is probably due to the homogeneity of the mixtures and the high formation of the phases of the polymerization process. Furthermore, when the temperature rises to a suitable range, most of the calcium carbonate ions in the mixture are decomposed, suggesting a decrease in the degree of reaction.

#### 3.8.3. FTIR Analysis

**Figure 16:**
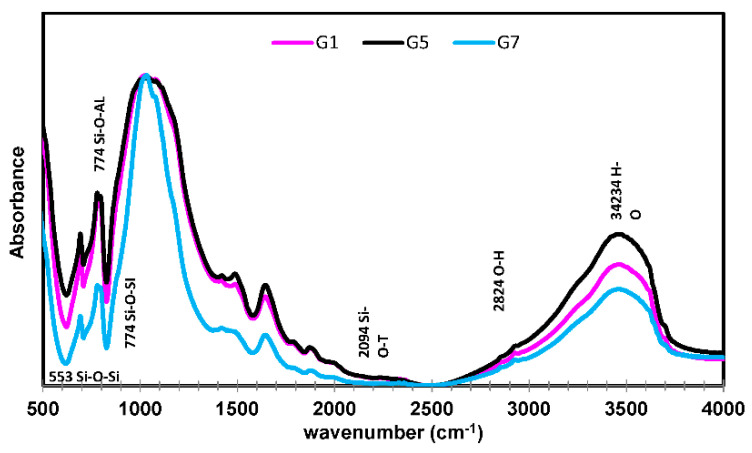
FTIR spectrum of UHPGC.

The UHPGC mixtures may be characterized using FTIR techniques, which provide information on the nanostructure and, therefore, the geopolymerization degree, as shown in Figure 16. Various bands and function groups created throughout the geopolymerization process explain the change in absorption frequency of the UHPGC mixtures. For the three mixtures (G1, G5, and G7), the initial peak was discovered in the range of 550–685 cm^−1^, which may refer to the symmetric stretching vibration of Si-O-Si. There were some differences in the transition, as shown in the diffraction patterns, demonstrating changes in the UHPGC matrix. The second peak took place in the wavenumber range of 747–810 cm^−1^, which could also refer to Si-O-Al bending vibration with aluminium (Al) [30]. The third vibration is observed in the region which is in the wavenumber region of 2100–2750 cm^−1^, matching with the stretching vibration of the functional group of Na_2_CO_3_. The final vibration, the bands at 3305–3590 cm^−1^, reveals that there are stretching vibrations of Si–OH bending. The high band at around 990–1150 cm^−1^ could be due to the symmetric and asymmetric stretching vibrations of Si–O and Al–O, which are formed during the dissolution of SiO_2_. Generally, the bands centered in these regions relate to the hydrate (N-A-S-H) gel in all UHPGC. As seen in the curves and some of the peaks formed, it is noted that the positive effect of the introduction of SF on the mixtures helped to improve the chemical reactions and the preferred bonds (Si-O-Si), as well as being identical to the highest ratio of Si/Al. On the other hand, there was a negative effect from the presence of fly-ash, which led to weak peaks. These findings are also in line with the results of the mechanical and previous studies [30].

## 4. Conclusions and Future Recommendations

In this study, experimental work was performed to achieve the early strength of the UHPGC with different KOH molarities, binder types, and curing temperatures. Hence, the following conclusions can be summarized below:

- It was found that every parameter considered in this study affected UHPGC’s early compressive strength. So, increasing KOH molarity decreases the setting times of mixtures, while decreasing KOH molarity increases the flowability of UHPGC mixtures.
- Adding silica fume up to 30% of the total aluminosilicate materials increased the early compressive strength of concrete up to 134 MPa in one day. Compressive strength decreased with the decrease in KOH molarity and curing temperature.
- Early strength compressive for all employed binders is enhanced by increasing the curing temperature from ambient curing to 100 °C. Early compressive strength for all binders is improved by increasing KOH molarity from 12 M to 16 M.
- UHPGC mixtures cured without heat gained strength gradually over time, but those cured at 60 and 100 °C for 8 and 24 h gained significant strength with time.
- The results indicate that the addition of fly ash to the UHPGC mixtures causes the early compressive strength to be decreased.
- In comparison to the control mix (100% GGBS) and the UHPGC mixture containing FA (90% GGBS + 10% FA), SEM micrographs demonstrate that the UHPGC combination with the optimal SF concentration (70% GGBS + 30% SF) obtains the densest microstructure. The optimum UHPGC mixture (G5) retained more than 95% of its weight, while the control mixtures (G1) lost 6% of their weight, according to TGA/DTA analysis.
- UHPGC enhanced with silica fume is a viable binder for high-strength concrete production under heat-curing conditions. Mixtures containing 70% slag, 30% silica fume, and 16 KOH molarity with heat curing are the best for early compressive strength and setting time comparable to the control mixture.
- As a recommendation, further research is needed on the durability of the early mechanical properties of UHPGC. The early strength of the one-part UHPGC needs more study. This will show a correlation between UHPGC’s early strength gain and aluminosilicate’s alkaline activation. The UHPGC study should also involve field application structural integrity.

## Figures and Tables

**Table 1:** Chemical composition of the ASM.

Composition (%).	SiO_2_	Al_2_O_3_	TiO_2_	Fe_2_O_3_	P_2_O_5_	MgO	CaO	MnO	K_2_O	Na_2_O
GGBS	41.66	13.96	0.68	1.59	0.06	5.67	34.53	0.39	0.97	0.49
SF	98.86	0.12	-	0.14	-	0.2	0.12	0.35	0.21	-
FA	62.19	28.18	2.37	4.54	0.47	0.49	0.58	0.07	1.05	0.06

**Table 2:** The parameters used for control of early compressive strength development.

Parameters	(%) of ASM			KOH Molarity (M)	Curing Temperature (°C)	W/ASM Ratio
	GGBS	SF	FA			
Values	60–100	0–30	10	12-14-16	Ambient, 60, and 100	0.211-.0221

**Table 3:** UHPGC mixtures proportions (kg/m^3^).

Series No.	Mixtures ID	ASM			Natural Sand	
		GGBS	SF	FA	Grade I	Grade II
Ref.	G1	865	-	-	383	766
Series 1	G2	800	51	-	383	766
	G3	744	102	-	383	766
	G4	699	154	-	383	766
	G5	644	205	-	383	766
Series 2	G6	865	-	66	383	766
	G7	713	51	66	383	766
	G8	648	102	66	383	766
	G9	583	154	66	383	766
	G10	518	205	66	383	766

**Table 4:** Critical molar ratios and W/ASM ratio of the UHPGC mixtures.

Mixtures ID	Molar Ratio			ASM/Sand	W/ASM At 16 M
	Na_2_O/SiO_2_	H_2_O/Na_2_O	SiO_2_/Al_2_O_3_		
G1	0.22	8.54	2.98	0.753	0.211
G2	0.21	8.56	3.56	0.741	0.214
G3	0.2	8.57	4.23	0.736	0.216
G4	0.19	8.59	5.03	0.742	0.214
G5	0.19	8.6	6.01	0.739	0.215
G6	0.21	8.54	2.85	0.735	0.216
G7	0.21	8.58	3.35	0.723	0.220
G8	0.2	8.59	3.93	0.718	0.221
G9	0.19	8.61	4.61	0.725	0.219
G10	0.18	8.62	5.42	0.721	0.220

**Table 5:** Research summary on early compressive strength (CS) of GPC.

Reference	Cementitious Material Used	Alkali Activator Used	Early CS (MPa)	Conclusion
Current study	GGBS, FA and SF	KOH + Na_2_SiO_3_	16–134	Increasing KOH molarity, enhancement of geopolymerization process, heat curing, and control the gradation of ASM and filler materials.
Nath et al. [48]	FA and GGBS	NaOH + Na_2_SiO_3_	40–63	Changing the Na_2_SiO_3_ to NaOH ratio from 1.5 to 2.5 resulted in a small loss in strength over time.
Assi et al. [49]	FA + SF	NaOH + Na_2_SiO_3_	93–106	SF-based activating solution increased CS compared to Na_2_SiO_3_-based activating solution.
Duan et al. [50]	Metakaolin	NaOH + Na_2_SiO_3_	20–50	At 3 h early curing, CS, and bond strengths approach 10 MPa and 0.6 MPa, respectively.
Shen et al. [51]	crushed waste brick	NaOH + Na_2_SiO_3_	5–31	At 6% alkali dosage and 3 days curing (70 °C, 30% Relative humidity), GPC’s CS reaches 31.1 MPa.
Alanazi et al. [37]	Metakaolin + GGBS	NaOH + Na_2_SiO_3_	21–58	A silicon dioxide/sodium oxide molar ratio of 10 accelerated geopolymerization and increased CS.
Assi et al. [42]	FA + SF	NaOH	30–68.5	NaOH affects CS. a 60–100% NaOH to binder ratio gives acceptable CS.
Li et al. [52]	GGBS	NaOH	16–41	GPC’s early CS was 120% higher than PCC’s (1 d) and its setting time was faster.
Elyamany et al. [41]	FA + SF + GGBS	NaOH+ Na_2_SiO_3_	35–45	Addition of GGBS and SF achieves better properties compared to fly ash only

**Table 6:** Average porosity of UHPGC mixtures.

Mixtures ID	G1	G2	G3	G4	G5	G6	G7	G8	G9	G10
Porosity (%)	2.77	3.27	2.61	2.57	1.89	3.73	3.65	3.03	2.87	2.81
Standard devi. (%)	±2.11	±4.61	±3.73	±1.61	±1.97	±2.44	±3.61	±2.11	±4.17	±3.05

## Data Availability

Data obtained as described.
